# Evaluation of analgesic, anti-inflammatory and CNS depressant activities of methanolic extract of *Lawsonia inermis* barks in mice

**Published:** 2014

**Authors:** Luthfun Nesa, Shirajum Munira, Shabnam Mollika, Monirul Islam, Habibullah choin, Aktar Uzzaman Chouduri, Nazmun Naher

**Affiliations:** 1 *Department of Pharmacy, Atish Dipankar University of Science and Technology, Dhaka, Bangladesh*; 2 *Department of Pharmacy, University of Rajshahi, Bangladesh *; 3 *Radiant Pharmaceutical Ltd, Tongi, Bangladesh*

**Keywords:** *Analgesic*, *Anti-inflammatory*, *Carrageenan*, *CNS depressant*, *Formalin induced pain Methanolic extract of Lawsonia inermis barks*, *Writhing*

## Abstract

**Objectives**: The study was carried out to assess the analgesic, anti-inflammatory, and CNS depressant activity of the methanolic extract of the *Lawsonia inermis *barks (MELIB).

**Materials and Methods:** Anti-inflammatory effects of MEBLI were studied using carrageenan-induced inflammatory method at the dose of 300 and 500 mg/kg b.wt., p.o. Analgesic activity was measured using acetic acid-induced writhing model and formalin-induced licking and biting in mice. The CNS depressant activity was evaluated by observing the reduction of locomotor and exploratory activities in the open field and hole cross tests at a dose of 300 and 500 mg/kg body weight.

**Results:** Statistical analysis showed that dose of 500 mg/kg exhibited higher analgesic activity against acetic acid-induced pain in mice than the standard drug diclofenac sodium. Furthermore, doses of 300 and 500 mg/kg caused higher percent of protection (91.16% and 95.03%, respectively) of licking and biting of formalin-induced mice than diclophenac sodium (70.72%). The Lawsonia inemis methanolic extract (300 and 500 mg/kg) also exhibited sustained inhibition (54.97% and 65.56%) of paw edema at the 4^th^ hour compared with standard indomethacin (74.17%). Besides, the plant extract also had significant (p<0.05) dose-dependent CNS depressant activity. **Conclusion**: this study recommends that the methanolic extract of *Lawsonia inermis *barks has significant analgesic, anti-inflammatory, and CNS depressant properties.

## Introduction

From the beginning of civilization, medicinal plants are part and parcel of human society to struggle diseases (Bandyopadhyay et al., 2002 ▶). There exist a plethora of knowledge, information, and welfares of herbal drugs in our earliest literature of Ayurvedic (Traditional Indian Medicine), Siddha, Unani, and Chinese medicine. The World Health Organization, 2003, reported that about 80% of the population of developing countries being incapable to afford pharmaceutical drugs rely on traditional medicines, mainly plant-based, to withstand their primary health care needs (Goyal et al., 2008 ▶). Herbal medicines are in great demand in the developed as well as developing countries for primary health care because of their wide biological and medicinal activities, higher safety margins, and lesser costs (Cragg et al., 1997 ▶; Padma, 2005). *Lawsonia inermis *Linn. (Family Lythraceae) is used all over the world and abundantly available in tropical and subtropical areas. Ancient history of India expresses its diverse uses and also shows appreciable role in Ayurvedic or natural herbal medicines (Lavhate and Mishra 2007 ▶).

*Lawsonia innermis* Linn (*L. inermis*) commonly known as henna/mehandi, have carbohydrates, proteins, flavonoids, tannins, as well as phenolic compounds, alkaloids, terpenoids, quinones, coumarins, xanthones, and fatty acids. This plant has been used for the treatment of epilepsy and jaundice, for dyeing gray hair, and for malignant ulcers (Dev, 2006 ▶). According to Ayurvedic Pharmacopoeia of India, the leaves are used in dysuria, bleeding disorder, and prurigo (Khare, 2007 ▶). Moreover, the leaves have a bitter bad taste with vulnerary and diuretic effects which are useful in the treatment of headache, hemicranias, lumbago, bronchitis, boils, ophthalmia, syphilitics, sores, amenorrhea, scabies, and diseases of the spleen and favor the growth of the hair (Kirkland and Marzin, 2003 ▶). Besides, the leaves are emetic and expectorant. The seeds are astringent to the bowels and antipyretic. The seeds are also a tonic for the brain. The flowers are funerary and an infusion cures headache. The bark is given in jaundice and enlargement of the spleen, also in calculus affection and as an alternative in leprosy and obstinate skin disease. In decoction form it is applied to burns and scalds (Devendra et al., 1977 ▶). Henna has antibacterial, antiviral, antimycotic, and antiparasitic activities (Dinesh and Subhasree 2009 ▶). 

Methanolic extract of henna at 0.25 and 3 (V/V) inhibits the growth of Malassezia (Fariba, 2010). The Lawsonia bark extract was found to possess fungistatic nature at its maximum inhibitory dilution of 1:30 (W/V) against both the test pathogens but became fungicidal at 1:10 (W/V). The extract showed a broad fungitoxic spectrum when tested against 13 ringworm fungi (Singh and Pandey 1989). The methanol extract of henna leaves at 1 mg/ml concentration displayed immune-stimulant action as indicated by promotion of T-lymphocyte proliferative responses (Makhija et al., 2011 ▶). The methanolic extract of henna reduced both hyperglycemia and inflammation significantly (Hasan et al., 2013 ▶). Additionally, the seeds had CNS depressant and anticonvulsant property acting via glycin receptor and the leaves showed a significant antioxidant property (Philip et al., 2011). According to the above-mentioned studies we aimed to determine the analgesic, anti-inflammatory, and CNS depressant effect of *Lawsonia inermis *barks methanolic extracts. 

## Material and Methods

**Plant material**

In the present investigation, the fresh barks of *Lawsonia inermis* were collected from the area of Rajshahi, Bangladesh and were identified by the experts of Bangladesh National Herbarium, Dhaka, with voucher specimen number 38586. The collected plant parts were dried for one week and pulverized into a coarse powder with the help of a suitable grinder. The powder was stored in an airtight container and kept in a cool, dark and dry place until analysis.

**Preparation of extracts**

About 150 g of powdered material was taken in a clean, flat bottomed glass container and soaked in 200 ml of 85% methanol. The container with its contents was sealed and kept for a period of 7 days accompanying occasional shaking and stirring. The whole mixture then underwent a coarse filtration by a piece of clean, white cotton material. Then, it was filtered through Whatman filter paper (Bibby RE200, SterilinLtd., UK). The obtained filtrate (methanol extract) was evaporated using a rotary evaporator. It rendered a gummy concentrate of reddish black color. The gummy concentrate was designated as a crude extract of methanol. The extract was transferred to a closed container for further use and protection.

**Animals**

The sixteen number of mice weighed between 20-25 g were used for each method. They were divided in to four groups, each cotainig four mice.The animals were obtained from the International Centre for Diarrheal Disease Research, Bangladesh (ICDDRB). All animals were kept under ambient temperature with 12 h light followed by a 12 h dark cycle. The animals were acclimatized for one week prior to actual experiments. The study was conducted following approval by the Institutional Animal Ethical Committee of University of Development Alternative, Dhaka, Bangladesh.

**Chemicals**

Diclofenac Sodium, Ibuprofen, and diazepam were obtained from Square Pharmaceuticals Ltd., Bangladesh and acetic acid was collected from Merck, Germany. Normal saline water (0.9%) NaCl was brought from Beximco Infusion Ltd. Bangladesh. BDH Chemicals Ltd provided Tween 80. Formalin, castor oil, carrageenan, and all other chemicals were of analytical grade. 

**Phytochemical Screening of the Extract**

The extract of *Lawsonia inermis *was subjected to qualitative analysis for the various phytoconstituents such as alkaloids, carbohydrates, glycosides, phytosterols, saponins, tannins, proteins, and flavonoids (Khandelwal KR, (2006) ▶. Data is provided in Table 1. 

**Analgesic activity**

*Acetic acid-induced writhing method*

The analgesic activity of the samples was studied using acetic acid-induced writhing model in rats (Winter et al., 1962). Test samples (300 and 500 mg/kg body weight), vehicle (1% Tween 80 in water), and diclofenac sodium (10 mg/kg) were administered orally 30 min before intraperitoneal administration of 0.7% acetic acid. Then, the mice were observed for specific contraction of the body, writhing, for the next 20 min (Achinta et al., 2007). Complete writhing was not always accomplished by the animal, because sometimes they started to give writhing but they did not complete it. This incomplete writhing was considered as half writhing. Accordingly, two half-writhings were taken as one full writhing. The number of writhes in each treated group was compared with that of a control group while diclofenac sodium (10 mg/kg) was used as a reference substance (positive control). 

The percent inhibition (% analgesic activity) was calculated by % inhibition = {(A-B) /A} X 100

Where, A = Average number of writhings of the control group and B= Average number of writhings of the test group.

*Formalin test*

The antinociceptive activity of the drugs was determined using the formalin test described by (Achinta et al., 2007). The control group received 5% formalin. Twenty μl of 5% formalin was injected into the dorsal surface of the right hind paw 60 min after administration of methanolic extracts of *Lawsonia inermis *barks (300 and 500 mg/kg, p.o.) and diclofenac sodium (10 mg/kg, p.o.). The mice were observed for 30 min after the injection of formalin and the amount of time spent licking the injected hind paw was recorded. The first 5 min post formalin injection was referred to as the early phase and the period between 15 and 30 min as late phase. The total time spent licking or biting the injured paw (pain behavior) was measured with a stop watch.

**Anti-inflammatory activity**

*Carrageenan-induced paw edema method*

The mice were divided into five groups each containing four mice. Acute inflammation was induced by injecting 0.1 ml of (1%) carrageenan into the plantar surface of the rat hind paw (Achinta et al., 2007). The MEBLI (300 and 500 mg/kg), normal saline (1 ml/kg), and ibuprofen at a dose of (10 mg/kg/i.p.) as referral agent were administered 30 min before carrageenan injection. The paw volume was measured at 1, 2 , 3 , and 4 h using a vernier caliper to determine the diameter of edema. The difference between the readings at time 1 h and different time interval was taken as the thickness of edema.

**CNS depressant activity**

*Hole cross test*

The method was carried out as described by (Sharma, 2010). A steel partition was fixed in the middle of a cage having a size of 30×20×14 cm^3^. A hole of 3 cm diameter was made at a height of 7.5 cm in the center of the cage. 

Sixteen animals were divided into four groups with four mice in each grou. Group I animals received vehicle (1% Tween 80 in water, 10 ml/kg, p.o.), animals of Group II received diazepam at 1 mg/kg body weight (p.o.) while Group III and Group IV were treated with 300 and 500 mg/kg body weight (p.o.) of the MEBLI. The number of passages of mice through the hole from one chamber to another was counted for a period of 3 min on 0, 30, 60, 90, and 120 min after oral administration of test drugs.

*Open field test*

The animals were treated as discussed above. The experiment was carried out according to the methods described by (Takagi, 1971). The floor of an open field of half square meter was divided into a series of squares each alternatively colored black and white. The apparatus had 40 cm height a wall. The number of squares visited by the animals was counted for 3 min, for 0, 30, 60, 90, and 120 min after oral administration of test drugs.

## Results

**Phytochemical screening of methanolic extracts of** ***Lawsonia inermis*** **Linn**

The result of the effect of *L. inermis* against acetic acid-induced writhing in mice is shown in Table 2. The *L. inermis* (300 and 500 mg/kg) dose dependently reduced acetic acid-induced abdominal constrictions and stretching. The reduction was significant (p<0.05) when compared with control. The effect of the extract was comparable to that of the standard drug, diclofenac sodium (10 mg/kg).

**Formalin induced hind paw licking in mice**

The result of the effect of the *L. inermis *against formalin-induced hind paw licking in mice is shown in Table 3. The *L. inermis* (300 and 500 mg/kg) pretreated animals showed a significant (p<0.05) dose-related reduction of the hind paw licking caused by formalin when compared with control and *L. inermis* treated with the dose of 300 mg/kg showed better activity when compared with standard (diclofenac sodium 10 mg/kg). 

**Carrageenan induced paw edema in mice**

The result of the effect of *L. inermis *on carrageenan-induced edema is shown in Table 4. The *L. inermis *exerted a significant (p<0.05) anti-inflammatory effect at the dose of 300 and 500 mg/kg and was comparable to that of the control group. The percentage inhibition activity of *L. inermis* (300 and 500 mg/kg) and standard (Ibuprofen) 10 mg/kg were found to be 54.97%, 62.25%, and 74.17%, respectively.

**CNS depressant activity**

*Hole-cross test*

Results of the hole-cross test of *L. inermis* is given in Table 5. They were statistically significant for all dose levels at 30, 60, and 90 min and followed a dose-dependent response. The depressing effect was most intense at dose 500 mg/kg.

*Open-field test*

Results of the open-field test of *L. inermis *is given Table 6. The *L. inermis *extract exhibited a decrease in the movements of the test animals at all dose levels. The results were statistically significant for all doses at 90 min and followed a dose-dependent response.

**Table 1:** Phytochemical screening of methanolic extracts of the bark of *Lawsonia inermis*

**Test **	**Glycoside**	**Phytosterol**	**Steroid**	**Saponin**	**Tannins**	**Flavonoid**
**Result**	**+**	**+**	**+**	**-**	**+**	**+**

Here, (+)ve = presence, (-)ve = absence

**Table 2:** Effects of the methanolic extract the bark of *Lawsonia  inermis* on acetic acid-induced writhing in mice

**Groups**	**Dose (mg/kg)**	**No. of writhing**	**% inhibition**
**Group I**	Vehicle	30.5(±1.136)	
**Group II**	10	7.75(±1.306)*	74.59
**Group III**	300	8.75 (±1.489)*	71.31
**Group IV**	500	5.25(±1.581)*	82.79

Values are mean±SEM, (n = 4);

* p<0.05 as compared with vehicle control (one way ANOVA followed by Dunnet’s test). Group I animals received vehicle (1% Tween 80 in water), Group II (Standard) received diclofenac sodium 10 mg/kg body weight, Group III and IV were treated with 300 and 500 mg/kg body weight (p.o.) of the methanolic extract of *Lawsonia inermis* barks, respectively.

**Table 3:** Effects of the methanolic extract of the bark of *Lawsonia inermis* on hindpaw licking in the formalin test in mice

**Groups**	**Dose (mg/kg)**	**Early phase (Sec)**	**% protection**	**Late phase (Sec)**	**% protection**
**Group-I**	Vehicle	36.5 ± 1.76	-	45.25 ± 1.69	-
**Group-II**	10	12.25 ± 1.31*	66.43	13.25 ±1.22*	70.72
**Group-III**	300	16.25 ± 1.31*	55.47	4.00 ± 1.22*	91.16
**Group-IV**	500	15.25 ± 1.97*	58.22	2.25±1. 62*	95.03

Values are mean±SEM, (n = 4);

* p<0.05 as compared with vehicle control (one way ANOVA followed by Dunnet’s test). Group I animals received vehicle (1% Tween 80 in water), Group II (Standard) received diclofenac sodium 10 mg/kg body weight, Group III and IV were treated with 300 and 500 mg/kg body weight (p.o.) of the methanolic extract of *Lawsonia inermis* barks, respectively.

**Table 4:** Effect of methanolic extract of the *Lawsonia inermis* barks on carrageenan-induced paw edema in mice

**Group**	**Dose**	**Oedema diameter(mm) 1h**	**2h**	**3h**	**4h**	**Inhibition (%) 1h **	**2h**	**3h**	**4h**
**Control**	Vehicle	4.6±0.907	4.35±0.71	4.25±0.56	3.78±0.44				
**Ibuprofen**	10 mg/kg	2.48±0.454*	1.9±0.46*	1.4±0.38*	0.98±0.31*	47.06	56.32	67.06	74.17
**MELI**	100 mg/kg	4.28±0.486*	2.45±0.56*	2.2±0.44*	1.7±0.6*	8.566	43.68	48.24	54.97
**MELI**	200 mg/kg	3.88±0.816*	2.5±0.82*	2.3±0.77*	1.43±0.72*	17.11	42.52	45.29	62.25

Probability values (calculated as compared with control using one way ANOVA followed by Dunnet’s test):

* p<0.05. All values are mean of individual data obtained from four mice (n=4), ± indicates standard error mean, Group III and IV were treated with 300 and 500 mg/kg body weight (p.o.) of the methanolic extract of *Lawsonia inermis* barks, respectively.

**Table 5:** Effect of methanolic extract of the *Lawsonia inermis* barks on hole cross test in mice

**Group**	**Dose**	**Number of Movements**				
		**0 min**	**30 min**	**60 min**	**90 min**	**120 min**
**Group-I**	10 ml/kg,	12 ± 1.716	16.75± 1.717	17.75±1.307	17.75± 1.307	19.25±1.489
**Group-II**	1 mg/kg,	15.75 ± 1.307	5.0± 1.107*	3.5± 1.136*	2.75± 0.978*	2.00 ± 0.904
**Group-III**	300 mg/kg	4.5 ± 1.443*	3.0±1.495*	2.25±1.622*	1.25± 1.376*	-
**Group-IV**	500 mg/kg	3.00 ± 1.351*	2.0± 1.0*	1.5± 1.136*	0.5±0.760*	-

Values are mean±SEM, (n = 4),

* p<0.05, Dunnet’s test as compared with vehicle control. Group I animals received vehicle (1% Tween 80 in water), Group II received diazepam 1 mg/kg body weight, Group III and Group IV were treated with 300 and 500 mg/kg body weight (p.o.) of the methanolic extract of *Lawsonia inermis* barks, respectively.

**Table 6:** Effect of methanolic extract of the bark of *Lawsonia inermis* on Open Field test in mice

**Group**	**Dose**	**Number of Movements**				
		**0 min**	**30 min**	**60 min**	**90 min**	**120 min**
**Group-I **	10 ml/kg	119 ± 1.469	115 ± 1.77	108.75±2.234	98.25±2.305	106.25± 1.942
**Group-II **	1 mg/kg	107.25± 2.510	72 ± 2.450*	49.5± 1.908*	21.25±1.608*	11.5±1.641
**Group-III **	300 mg/kg,	237.5 ± 5.00*	108.5± 4.533	69.75±5.019*	30±4.272*	-
**Group-IV **	500 mg/kg,	160.5± 8.612	94.75± 5.659	88.75±5.475	49±6.026*	-

Values are mean±SEM, (n = 4),

* p<0.05, Dunnet’s test as compared with vehicle control. Group I animals received vehicle (1% Tween 80 in water), Group II received diazepam 1 mg/kg body weight, Group III and Group IV were treated with 300 and 500 mg/kg body weight (p.o.) of the methanolic extract of *Lawsonia inermis* barks, respectively.

## Discussion

The extensive survey of literature revealed that *L. inermis* is highly regarded as a universal panacea in the herbal medicine with diverse pharmacological activity spectrum. A drug development program should be undertaken to develop modern drugs with the compounds isolated from henna (Gagandeep et al., 2010 ▶). Acetic acid-induces pain by enhancing levels of PGE_2 _and PGF_2α_ (Deraedt et al., 1980 ▶) at the receptors of the peritoneal cavity (Bently et al., 1983 ▶; Lee and Choi, 2008 ▶), which means that the acetic acid acts indirectly by increasing the release of endogenous mediators leading to stimulation of the nociceptive neurons which are sensitive to most of the non-steroidal anti-inflammatory drugs. 

Two different doses (300 and 500 mg/kg b.w.) of crude extract of bark of *L. inermis *showed significant (71.31% and 82.79%) analgesic action while higher doses (500 mg/kg) were found to exhibit more analgesic activity against acetic acid-induced pain in mice compared with the reference drug diclofenac sodium. This result suggests the involvement of peripheral mechanisms of analgesia. 

The formalin test is another important model of analgesic which is better related to clinical pain (Tjolsen et al., 1992; Ghanadi et al., 2005 ▶). This method elucidates central and peripheral activities. Formalin-induced nociception is biphasic in which first phase involves direct stimulation of sensory nerve fibers representing neuropathic pain and second phase involves inflammatory pain mediated by prostaglandin, serotonin, histamine, bradikinin, and cytokines such as IL-1β, IL-6, TNF-α, eicosanoids, and NO (Murrey et al., 1988 ▶; Watkins et al., 1997; Chichorro et al., 2004 ▶; Hunskar and Hole 1987 ▶; Moore et al., 1991 ▶; Santodomingo-Garzon et al., 2006). *L. inermis *showed inhibition at the second phase of formalin-induced nociception in mice and this inhibition was higher than the standard diclofenac sodium. The *L. inermis* was found to exhibit analgesic effect by reducing hypernociception induced by bradykinin and cytokines (TNF-α,IL-1β) and the release of IL-1β and PGE_2 _in paw skin induced by polysaccharide (Gupta et al., 1971 ▶). Besides, the extract at the dose of (300 and 500 mg/kg) caused higher percent of protection (91.16% and 95.03%, respectively) against licking and biting induced mice than standard, diclophenac sodium (70.72%) and in this test, the inhibition over licking response of the MEBLI at the late phase demonstrate signifying analgesic effect of the extract on the formalin test.

Carageenan–induced paw edema has been commonly used as an experimental animal model for acute inflammation and is believed to be biphasic in which the early phase (1-2) of the carageenan model is mainly mediated by histamine, serotonin, and increased synthesis of prostaglandins in the damaged tissue surroundings and the late phase is sustained by prostaglandin release and mediated by bradykinin, leukotrienenes, polymorphonuclear cells, and prostaglandins produced by tissue macrophase (Gupta et al., 2006 ▶; Gupta et al.,1993 ▶). 

Isoplumbagin and lawsaritol isolated from stem bark and root of *L. inermis *L. showed anti-inflammatory activity against carrageenan-induced paw oedema in mice. The compounds phenylbutazone, isoplumbagin, and lawsaritol isolated from *L. inermis *at the oral dose of 100 mg/kg exhibited 61, 60, and 40 percent inhibition in comparison with controls (Gupta et al., 1993 ▶). Besides, in our study, the crude methanol extract (300 and 500 mg/kg) of *L. inermis *exhibited significant and sustained inhibition (54.97% and 65.56%) of paw edema at the 4^th^ hour while the standard indomethacin reported 74.17% inhibition. The possible mechanism of the observed anti-inflammatory activity might be its ability to reduce the release of histamine, serotonin or kinin-like substances or biosynthesis of prostaglandins which is consistent with the test of analgesic activity.

Locomotor activity considered as an increase in alertness and decrease in locomotor activity indicated sedative effect (Kolawole et al., 2007 ▶). The current study examined some neuropharmacological activities of methanolic extract of *L. inermis*. The plant extract possessed central nervous system depressant activity as indicated by the decrease in exploratory behavior in mice. Moreover, the study of locomotor activity, as measured by hole cross and open field tests, showed that extracts of the *L. inermis* barks (300 mg/kg and 500 mg/kg) decreased the frequency and the amplitude of movements. Since, locomotor activity is a measure of the level of excitability of the CNS (Mansur et al., 1980 ▶), this decrease in spontaneous motor activity could be attributed to the sedative effect of the plant extracts (Rakotonirina et al., 2001; Ozturk et al., 1996 ▶).

 The locomotor activity lowering effect was evident in the 2^nd^ observation (30 min) and continued up to a 4^th^ observation period (90 min). Maximum depression of locomotor activity was observed from the 3^rd^ (60 min) to 4^th^ (60 min) observation period. Gamma-amino-butyric acid (GABA) is the major inhibitory neurotransmitter in the central nervous system. Different anxiolytic, muscle relaxant and sedative-hypnotic drugs elucidate their action through GABA, therefore it is possible that extracts of *L. inermis *may act by potentiating GABAergic inhibition in the CNS via membrane hyperpolarization which leads to a decrease in the firing rate of cortical neurons in the brain or may be due to direct activation of GABA receptor by the extracts (Bhattacharya et al., 1997 ▶). 

Many researchers suggest that plants containing flavonoids, saponins, and tannins are useful in many CNS disorders (Verma et al., 2010). Earlier investigations on phytoconstituents and plants suggest that many flavonoids and neuroactive steroids were found to be ligands for the GABA receptors in the central nervous system which led to assuming that they can act as benzodiazepine-like molecules (Ghanshyam et al., 2013 ▶). Therefore, these phytoconstituents may be responsible for *Lawsonia inermis *CNS depressant activity. In conclusion, the bark extract *Lawsonia inermis *possesses remarkable antinociceptive, anti-inflammatory, and CNS depressant activities. The present work was a preliminary effort which requires further detailed investigation including characterization of active compounds and preformulation studies for development of a potential dosage form.
